# Primary Aldosteronism and Cognitive Dysfunction: A Case-Control Study

**DOI:** 10.3390/jcm14134618

**Published:** 2025-06-30

**Authors:** Jakov Herceg, Gorana Vukorepa, Sandra Karanović Štambuk

**Affiliations:** 1 School of Medicine, University of Zagreb, 10000 Zagreb, Croatia; herceg.jak@gmail.com; 2 Department of Neurology, Klinik Landstrasse, 1030 Vienna, Austria; gorana.vukorepa@gesundheitsverbund.at; 3 Department of Internal Medicine, Division of Nephrology, Arterial Hypertension, Dialysis and Transplantation, University Hospital Center Zagreb, 10000 Zagreb, Croatia

**Keywords:** cognitive dysfunction, essential hypertension, mini-mental state examination, montreal cognitive assessment, primary aldosteronism

## Abstract

**Background**: Primary aldosteronism is characterized by elevated aldosterone levels, leading to adverse effects such as hypertension, hypokalaemia and increased risk for cardiovascular and cerebrovascular events. Aldosterone impacts the central nervous system by promoting vascular remodelling and oxidative stress, potentially impairing cognitive function. The presence of mineralocorticoid receptors in the hippocampus, a key region for cognition, further suggest a link between primary aldosteronism and cognitive dysfunction. This study aims to further explore the association between hyperaldosteronism and cognitive impairment. **Methods**: In this pilot study we examined 15 individuals with primary aldosteronism and arterial hypertension alongside 15 age- and sex-matched controls with essential hypertension, all free of previous cerebrovascular events. Clinical and archival laboratory data were obtained. Cognitive function was assessed using the Mini-Mental State Examination and Montreal Cognitive Assessment. **Results**: Participants with primary aldosteronism had higher blood pressure values, longer duration of hypertension, lower serum potassium levels and higher 24 h urine albumin excretion rate compared to controls. Comorbidities, other characteristics and laboratory values were comparable across the two groups. No differences were observed in Mini-Mental State Examination scores, but Montreal Cognitive Assessment scores were significantly lower in the primary aldosteronism group (25.1 ± 2.2 vs. 27.1 ± 2.2, *p* = 0.021). Trends of poorer performance in language and attention/executive function domains were noted in primary aldosteronism individuals, as well as a higher number of pathological Montreal Cognitive Assessment scores (7 vs. 3). No significant correlations were found between cognitive test results and aldosterone concentrations or blood pressure in primary aldosteronism group. However, importantly, multiple regression analysis showed that aldosterone levels have a significant impact on Montreal Cognitive Assessment test, independent of blood pressure or duration of hypertension. **Conclusions**: This study supports an association between hyperaldosteronism and cognitive dysfunction, underscoring the need for more active detection and targeted treatment of primary aldosteronism. These findings warrant further research in larger cohorts to better elucidate this relationship.

## 1. Introduction

Cognitive disorders are characterized by impaired mental functions such as memory, attention, perception, language, thinking, and problem-solving abilities, unrelated to the normal aging process. These deficits might start interfering with independence in daily activities, placing a heavy burden, not only on the patients, but also their families and the entire healthcare system. Globally, the number of people living with dementia is expected to increase to 115.4 million by 2050 [1]. Consequently, identifying and understanding potential risk factors has become a critical focus in contemporary medical research.

Arterial hypertension is the biggest modifiable risk factor for the development and progression of mild cognitive impairment (MCI) and vascular dementia [2]. Recent research highlights its role in the pathogenesis of Alzheimer’s disease as well [3]. The underlying cause of cognitive impairment in individuals with high blood pressure is damage to small subcortical blood vessels. Exposure of the brain’s small blood vessels to elevated blood pressure, pulsating pressure and flow, leads to microvascular damage [4], resulting in white matter damage, which appears on magnetic resonance imaging (MRI) as hyperintensities known as white matter lesions (WML) and cortical dysfunction [5]. Hypertension causes brain function impairment through acute and silent cerebral ischemia and haemorrhage, accelerated brain tissue loss, rarefaction of cerebral microvasculature, endothelial cell dysfunction, disruption of the blood-brain barrier, and neuroinflammation which affects the pathological accumulation of beta-amyloid, the main pathohistological finding in Alzheimer’s [6].

Aldosterone is a mineralocorticoid hormone derived from the adrenal cortex and a part of the renin-angiotensin-aldosterone cascade. It is a key factor in the multifactorial regulation of salt, potassium and blood pressure in the body, primarily acting on the nephron’s distal tubule. Hyperaldosteronism, on the other hand, has negative effects, causing arterial hypertension but also promoting inflammation and fibrosis by binding to local mineralocorticoid receptors (MR) in tissues, particularly in the heart, smooth muscle cells of blood vessels and kidneys, thus enhancing damage in addition to high blood pressure itself. MR have also been found in the central nervous system (CNS) and aldosterone is being investigated in the context of cognitive function [7].

Primary aldosteronism (PA) represents inappropriately high, autonomous, aldosterone secretion, and is the most common cause of secondary hypertension, affecting >5–10% of hypertensive patients [8]. The effects of aldosterone are the subject of several studies and represent a potential new therapeutic direction in the treatment of neurocognitive disorders [9]. Studies have suggest that increased aldosterone levels are a significant risk factor in the development of stroke, one of the leading causes of cognitive dysfunction [10]. Additionally, positive effects of aldosterone inhibitors on preserving cognitive function and also preventing cerebrovascular events were observed [9].

Cognitive impairment in hyperaldosteronism is considered to be caused by the phenomenon known as aldosterone-mediated cerebrovascular remodelling. MR activation promotes vascular inflammation and oxidative stress, resulting in disrupted blood flow regulation in the brain [11]. Aldosterone also has a strong effect on the development of atherosclerosis [12]. Thus, high serum aldosterone level seems to enhance the main pathophysiological mechanisms in the development of vascular dementia. Adding the fact that MRs are abundantly expressed in the brain, particularly in the hippocampus which plays a crucial role in human cognition, it is clear why there is a growing need to discover the extent of the impact of aldosterone on mental functions. Additionally, a correlation has been proven between plasma aldosterone levels and the presence of WML on MRI, which is a reliable indicator of the existence of cerebral damage that leads to cognitive dysfunction [13].

A possible connection between PA and the development of anxiety disorders, depression and sleep disturbances has also been observed, all of which are well-known risk factors for impaired cognitive performance, underscoring additional potential negative effects of hyperaldosteronism [14].

Still, the currently available literature data on the direct and independent effect of PA on cognitive function remains inconsistent [15,16].

The aim of this study was to compare cognitive performance between individuals with PA and arterial hypertension and those with essential hypertension in order to assess whether patients with PA and high blood pressure exhibit more pronounced cognitive dysfunction compared to essential hypertension controls.

## 2. Materials and Methods

To investigate the influence of PA on cognitive abilities, we conducted a case-control study in the University Hospital Center (UHC) Zagreb, Croatia, from 2022 to 2023. The research was approved by the Ethics Committee of the UHC Zagreb (ethical approval code: 02/013-JG) and was performed in accordance with the ethical standards of the Declaration of Helsinki. All participants provided written informed consent prior to enrollment.

### 2.1. Patients

The case group included participants with biochemically confirmed PA [8] and consequent arterial hypertension, diagnosed at the Division of Nephrology, Arterial Hypertension, Dialysis, and Transplantation of UHC Zagreb. These individuals had not yet undergone adrenalectomy or treatment with MR antagonists. Each PA participant was matched with a control of the same sex and age but with essential hypertension (EH). The study included 15 participants with PA and 15 controls with EH, comprising 6 women and 9 men in each group. PA participants were consecutively diagnosed and enrolled, while controls were selected from the Ambulatory Blood Pressure Measurement Clinic, matched to the PA’s group characteristics. Individuals with a history of stroke or other comorbidities that could affect cognitive function, such as depression, anxiety, transitory ischemic attacks, as well as patients with secondary forms of arterial hypertension apart from PA were excluded from the study.

### 2.2. Data Collection

Data that were collected for all participants included age, sex, years of education and education level, comorbidities, list of current medications, smoking status and duration of arterial hypertension. Waist circumference, height and weight were measured, and body mass index (BMI) was calculated using the formula: weight (kg)/height (m)^2^. Blood pressure was measured according to the European Society of Hypertension guidelines [17], using an oscillometric device with an appropriate cuff size. The mean of the last two measurements of both systolic (SBP) and diastolic (DBP) blood pressure values was used for statistical analysis. Mean arterial pressure (MAP) was calculated using the formula: (SBP − DBP)/3 + DBP, (mmHg). Left ventricular hypertrophy (LVH) was determined from participants’ archival electrocardiograms. Archival laboratory data were obtained for all participants, including haemoglobin, haematocrit, serum creatinine, estimated glomerular filtration rate calculated by CKD EPI equation (eGFR CKD EPI), liver function tests (alanine transaminase, aspartate transaminase, alkaline phosphatase, bilirubin and gamma-glutamyltransferase), serum and urine electrolytes (serum sodium, potassium, chloride, calcium, phosphorus levels and sodium and potassium excretion values from 24 h urine collections), fasting blood glucose, haemoglobin A1c (HbA1c), serum uric acid levels, total cholesterol, LDL cholesterol and lipoprotein a [Lp(a)] concentrations, 24 h urinary albumin excretion rate and finally, serum aldosterone concentrations, plasma renin activity (PRA) and their ratio (ARR). All laboratory diagnostics as well as electrocardiograms were performed as part of the arterial hypertension evaluation shortly prior to inclusion in the study. To further analyze hypertension severity between two groups, participants were classified into two levels of severity: controlled and uncontrolled, with the latter defined by office blood pressure values ≥ 140/90 mmHg despite antihypertensive treatment.

### 2.3. Cognitive Function Assessment

Cognitive function was assessed using the Mini-Mental State Examination and the Montreal Cognitive Assessment.

Mini-Mental State Examination (MMSE) is a widely used screening tool for examining cognitive function [18]. It consists of eleven items and evaluates several cognitive domains including orientation (temporal and spatial), memory (registration and recall), attention/concentration, language (verbal and written) and visuospatial function, usually taking 5–10 min to complete. The results of the test are to be adjusted for age and years of education. The maximum score is 30 points; higher scores indicate better cognitive performance. A cut-off score of 24 points is commonly used to indicate cognitive impairment, with scores below this threshold considered indicative of cognitive dysfunction [19]. MMSE has pooled sensitivity of 81% and a specificity of 89% when distinguishing patients with dementia from cognitively normal individuals [20], while in identifying patients with MCI, MMSE exhibits a pooled sensitivity of 62.7% and a specificity of 63.3% [19]. Accordingly, the test is most commonly indicated for screening moderate to severe cognitive impairment, particularly in the elderly population, and is frequently used in both clinical and research settings to monitor cognitive status over time [21].

In this study, MMSE results were expressed as a ratio of the observed score to the expected score, adjusted for the participant’s age and level of education.

The Montreal Cognitive Assessment (MoCA) is a brief and sensitive instrument that evaluates a broad range of cognitive domains, including attention and concentration, executive functions, memory, language, visuospatial abilities, abstract thinking, calculation and orientation, taking up approximately 10 min to complete. The total score ranges from 0 to 30, with higher scores also demonstrating better cognitive performance. According to the established guidelines, a cut-off score of 26 is commonly used and scores below 26 are considered indicative of cognitive impairment [22]. Additionally, one point is added for participants with 12 or fewer years of formal education, as per test instructions [22]. In comparison with MMSE, MoCA shows higher sensitivity for MCI detection. MoCA exhibits a pooled sensitivity of 91% and a pooled specificity of 81% for identifying dementia, and a pooled sensitivity of 89% and a pooled specificity of 75% for identifying MCI [20]. Accordingly, MoCA is particularly useful for detecting subtle deficits and is preferred over the MMSE in cases of suspected early-stage cognitive decline, especially among highly educated or high-functioning individuals [22,23].

In our study, MoCA results were reported as absolute values, and participants were categorized into two groups based on their scores: normal or pathological.

### 2.4. Statistical Analysis

Statistical analysis was performed using JASP version 0.19.3. The distribution of scale variables was assessed using the Shapiro-Wilk test, distribution plots and Q-Q diagrams. For variables that did not follow normal distribution, data were presented as median and range (minimum and maximum values), and the non-parametric Mann-Whitney test was used to compare groups. For normally distributed data means and standard deviations were reported. When normally distributed data showed unequal variances between groups, Welch’s *t*-test was used; otherwise, Student’s *t*-test was applied. Differences in variance between groups were tested using either Brown-Forsythe or Levene’s test, depending on the distribution. Correlations between variables were assessed using Pearson’s correlation for parametric distributions and Kendall’s Tau B correlation for non-parametric data distributions due to the small sample size and many tied ranks. Fisher’s exact test was used to analyze the proportions of nominal variables. Multiple linear regression analysis was utilized to assess the association between plasma aldosterone levels and cognitive function, adjusting for potential confounders including blood pressure. No power analysis was conducted, as this study was exploratory in nature and treated as a pilot. Statistical significance was set at *p* < 0.05.

## 3. Results

The study included 15 cases with PA and arterial hypertension and equal number of participants with EH, matched for age and sex. Mean age of participants was 53 ± 7.2 years in both groups.

The groups differed significantly in SBP, DBP, and MAP as well as in serum potassium, aldosterone concentration, PRA and ARR, with higher blood pressure values, aldosterone concentrations and ARR levels, together with lower potassium concentrations and lower PRA, measured in the PA group. Although a greater proportion of individuals in the PA group had uncontrolled hypertension (13 participants, 86.6%), compared to the control EH group (8 participants, 53.3%), this difference did not reach statistical significance (*p* = 0.108). No statistically significant differences were found in the total number of medications used by participants within the two groups or in the number of antihypertensive drugs; however the PA group exhibited significantly longer duration of arterial hypertension than individuals with EH [10 (1–22) vs. 3 (1–17) years, *p* = 0.033].

There were no significant differences in comorbidities or the number of smokers between the two groups, as well as in BMI or waist circumference. In terms of laboratory parameters tested, apart from aforementioned hormones and potassium, the groups did not differ significantly in haemoglobin, haematocrit, kidney and liver function tests, serum sodium, chloride, calcium, phosphorus, uric acid, lipid levels including Lp(a), fasting blood glucose and HbA1c, 24 h urine sodium and potassium excretion. There was significantly higher 24 h albumin excretion rate in the PA group in comparison with EH (22 (5–330) vs. 8 (5–39) mg/dU, *p* = 0.039). The groups did not differ in the number of individuals with LVH detected on electrocardiogram.

No significant differences were observed between the groups in terms of educational level.

Participants’ characteristics and between-group statistical comparisons are summarized in Table 1a–c, as well as Supplementary Table S1.

Among participants with PA, the median MMSE score was 27 (range: 22–30), while in the control group with EH, the median score was 29 (range: 27–30), representing a statistically significant difference as determined by the Mann-Whitney U test (U = 66.5, *p* = 0.050). However, since the MMSE scores are influenced by years of education, and our cases and controls were not initially matched by education levels, despite lack of difference in average education level among groups, the obtained MMSE scores were adjusted by dividing each individual’s total score with their maximum possible score corrected for age and education. These adjusted values, referred to as MMSE success percentages, were then compared between the two groups. The average MMSE success percentage in the PA group was 98.3 ± 8.2%, compared to 101.2 ± 2.9% in the control group—a difference that was no longer statistically significant, as was determined by the Welch test, t(17.42) = 1304, *p* = 0.209.

Cognitive function assessed using the MoCA test revealed a statistically significant difference: the PA group had an average score of 25.1 ± 2.2, while the control EH group scored 27.1 ± 2.2, with *p* = 0.021. The results are presented in Table 2.

There was only 1 participant with a pathological MMSE test (below 24) in the PA group, while there were no individuals with EH who had a pathological MMSE test score. A pathological MoCa test (below 26) was present in 7 individuals with PA and in 3 patients in the control EH group, however this difference did not reach statistical significance (OR = 3.5, *p* = 0.2451).

In the further analysis, we individually examined the cognitive domains assessed by the MoCA test, which included visuospatial abilities, naming, attention and concentration, language, abstract thinking, delayed recall, and orientation. No significant differences were found in these subdomains between individuals with PA and controls with EH. However, there was a trend toward lower scores in the visuospatial/executive abilities and language domains in the PA group, as presented in Table 3.

Next, we examined the correlation between elevated aldosterone levels and cognitive function in participants with PA. Kendall’s tau-b correlation test did not reveal any statistically significant associations between either serum aldosterone concentration or the aldosterone-to-renin activity ratio and the results of the MMSE and MoCA tests. Furthermore, serum sodium and potassium levels, systolic and diastolic blood pressure, MAP, duration of hypertension, BMI and waist circumference, were not significantly correlated with cognitive function test scores in individuals with PA. No significant association was found between the number of medications used and cognitive testing results either. However, albuminuria showed significant negative correlation with the results of the MMSE in PA group and fasting blood glucose negative correlation with results of the MoCA test. Correlation analysis results are presented in Table 4.

Finally, because we observed significant differences in MoCA scores between PA and EH groups, and these groups were not initially matched for blood pressure values and some other factors potentially affecting cognitive function, we performed multiple regression analysis. This allowed us to isolate the impact of aldosterone on cognitive function independently of blood pressure and to test whether other variables also influence cognitive outcomes. The results showed that serum aldosterone levels as well as 24 h urine albumin excretion rate are statistically significant predictors of cognitive function (*p* < 0.05), while SBP, duration of hypertension, years of education, serum sodium and fasting blood glucose levels, PRA and ARR were statistically insignificant in our cohort (≥0.05). The results are shown in Table 5.

## 4. Discussion

Cognitive impairment significantly contributes to global mortality and disability [24]. Even though arterial hypertension is the biggest modifiable risk factor for the development and progression of MCI and vascular dementia, additional risk factors keep emerging. Growing evidence highlights the impact of aldosterone on cognitive function as well [25]. PA, characterized by inappropriately high aldosterone concentrations, causes numerous adverse effects including arterial hypertension, hypokalemia, and organ damage to the kidneys, heart, and vasculature. Studies have demonstrated a higher incidence of cardiovascular and cerebrovascular events in individuals with PA compared to those with EH, matched for age, sex and blood pressure values [8]. Given aldosterone’s role in cerebrovascular remodelling, promotion of vascular inflammation and oxidative stress, and disruption of cerebral blood flow regulation, as well as the presence of MR in the hippocampus, a region critical for cognitive function, we conducted a study on cognitive function in 15 targeted treatment naive individuals with PA and arterial hypertension, and 15 age and sex-matched controls with EH, all free of previous cerebrovascular events.

The groups did not differ significantly in years of education, one of the key factors in cognitive function testing. Similarly, there were no differences in waist circumference or BMI, both relevant considering the link between adipose tissue and aldosterone [11]. Also, groups were comparable in the number of smokers as well as in participants’ comorbidities, red blood cell counts, electrolytes’ concentrations apart from serum potassium, kidney and liver function tests, glucose homeostasis tests and lipid levels—all of which may also potentially impact cognitive function.

In this cohort unmatched for blood pressure values, the PA individuals had higher blood pressure values and lower serum potassium concentrations than individuals with EH, reflecting aldosterone’s effect on volume and sodium retention, and potassium excretion. Also, PA group had longer duration of hypertension, suggesting a probable delay in diagnosis—a challenge encountered worldwide [26,27], which is of particular importance as it increases the morbidity and mortality in these individuals. A potential step towards addressing this significant issue is reflected in the new ESC guidelines, which recommend considering screening for PA in all individuals with confirmed arterial hypertension [28].

Interestingly, there was no significant difference in the number of antihypertensive medications used by the two groups—both had a median of three. However, it should be noted that in PA patients, despite the use of three antihypertensives, blood pressure was not sufficiently regulated, whereas the control group had high normal values with therapy. These findings align with expectations, as PA patients had not yet been treated with adrenalectomy or MR antagonists.

Participants in the PA group had higher values of 24 h urine albumin excretion rate (22 (5–330) vs. 8 (5–39) mg/dU, *p* = 0.039) with comparable eGFR in both groups. Although albuminuria values in PA individuals remained within the mildly increased range, these levels still suggest an elevated cardiovascular risk, as demonstrated in the Framingham Heart Study [29]. Interestingly no differences were found in the number of participant with LVH between the groups, likely due to the moderate sensitivity and specificity of ECG and the absence of imaging data.

Cognitive function was assessed using two screening tools: the MMSE and MoCa. As already mentioned, one should have in mind higher sensitivity and specificity of MoCA for detecting mild cognitive impairment compared to MMSE [20]. MMSE performance showed a trend toward lower scores in the PA group, however the difference was not statistically significant. Importantly, the MoCa scores were significantly lower among PA patients suggesting negative effect of primary aldosteronism on cognitive function. Although we did not observe statistical significant difference in MoCa test results across specific cognitive domains, a trend toward lower performance in language and attention/executive function was noted in the PA group, the latter one important in terms of subcortical disease and development of vascular dementia [30]. Also, a trend toward more individuals in the PA group with pathological MoCa score was observed in comparison with EH (7 vs. 3, *p* = 0.2451). The lack of statistical significance may be attributed to the small sample size or insufficient duration of aldosterone exposure to manifest measurable cognitive decline in all participants.

To date, only a limited number of studies have investigated the impact of PA on cognitive function, particularly in comparison to the effects observed in patients with EH. Engler et al. examined the effects of elevated aldosterone in patients with newly diagnosed PA [15]. Using various psychiatric and neuropsychological tests, they assessed mood, quality of life, sleep, and cognitive functions. While depression, anxiety, and reduced quality of life were observed, no specific impact of high aldosterone levels on cognitive performance was found. The main limitations of this study were small sample size and relatively young age of participants [15]. Yagi et al. found that individuals with primary hypertension and relatively high—though normal—aldosterone levels had reduced cognitive performance on the MMSE which significantly improved after six months of MR antagonist therapy, suggesting a possible link between aldosterone and cognitive function [9]. More recently, a nationwide cohort study by Hong et al. revealed a potential association between PA and an increased dementia risk compared to EH [16].

In our study, we found no significant correlations between aldosterone levels or the aldosterone-to-renin ratio and cognitive test results in the PA group. This may be attributable to the small sample size, but could also reflect a threshold effect; that is, there may be a level of aldosterone above which the risk of cognitive impairment does not increase further, or all participants may already be exposed to a sufficiently high level such that the effect is saturated.

We also did not observe significant correlations between cognitive scores and systolic and diastolic blood pressure in the PA group. It is possible that increasing the number of participants would yield a significant correlation for both systolic and diastolic pressure. It is also worth noting that recent research findings, such as those by Melgarejo et al., suggest that blood pressure variability rather than absolute blood pressure levels may play a more critical role in cognitive decline [31], as well as duration of hypertension, namely several studies including Honolulu-Asia Aging Study demonstrated cognitive decline in people aged ≥ 70 when BP was elevated in the fourth or fifth decade of life [32], meaning middle age is maximal risk period and takes few decades to express cognitive impairment after exposure to elevated blood pressure. On the other hand, we did find significant negative correlation of 24 h urine albumin excretion rate and MMSE scores, probably due to detection of less subtle changes in cognitive function by MMSE when other target organ damage may be present too. Also, we found statistically significant negative correlation between fasting blood glucose and MoCA test results pointing out on increased glycaemia as a known risk factor for cardiovascular events as well as cognitive impairment [33].

Finally, multiple regression analysis confirmed a significant negative impact of aldosterone on MoCA test results, i.e., cognitive function, independent of blood pressure levels or duration of hypertension. Albuminuria was also identified as a significant predictor of lower MoCA scores, suggesting a probable shared microvascular pathology in both the kidney and the brain as previously reported by Georgakis et al. [34].

Overall, the results of this study support a direct association between PA and cognitive function, beyond the effect of blood pressure itself. Given the results of multiple regression analysis, although based on a small sample number, we can attribute cognitive impairment to PA independently of hypertension. These findings highlight the importance of more active detection and targeted treatment of primary aldosteronism, as well as potential implementation of routine cognitive screening in these patients.

The major limitations of the study include small sample size and the discrepancy in blood pressure values between the groups. In order to overcome the latter limitation, multiple regression analysis was performed, however a more appropriate study design would have included groups with comparable blood pressure values. It would definitely be beneficial to validate the results on a larger cohort of participants fully matched for blood pressure values. Additional limitations include lack of data on prior blood pressure control, as well as the absence of data on pH and bicarbonate levels, which are unfortunately unavailable. Ambulatory blood pressure monitor data might show better insight into blood pressure values than office blood pressure measurement, also identifying blood pressure variability and dipping pattern that might impact cognitive performance [35]. Furthermore, the MMSE and MoCA are effective screening tools for cognitive functions, but they are not sufficient for a comprehensive cognitive assessment. A broader battery of neuropsychological tests is recommended for a more detailed evaluation.

In summary, extending this pilot study to a larger cohort matched for blood pressure values and inclusion of additional parameters would be of considerable interest. The use of MRI to assess WML has shown promise in identifying early signs of neurocognitive disorders [36,37]. Notably, study by Yuan et al. has established a connection between high plasma aldosterone concentrations and the development of WML [38]. Therefore, in addition to a larger sample size, matching blood pressure values between the groups, a broader battery of cognitive function tests, additional laboratory data, and potentially the inclusion of ambulatory blood pressure monitor data, the MRI-based analysis of the presence, location, size and number of WMLs, may be a valuable tool for further investigating the relationship between PA and cognitive dysfunction.

## 5. Conclusions

Our study demonstrated that aldosterone exerts an effect on cognitive function, as assessed primarily by the more sensitive Montreal Cognitive Assessment test, independently of blood pressure levels, duration of hypertension, or prior cerebrovascular events. Consequently, these patients, in addition to an elevated risk of cardio/cerebrovascular events, are also at increased risk for cognitive impairment, which may ultimately progress to dementia. Worldwide, both the proportion of diagnosed cases and the average time to diagnosis of primary aldosteronism indicate significant room for improvement. We underscore the importance of timely screening for PA as well as targeted treatment. Furthermore, we highlight the potential value of implementing routine screening for cognitive dysfunction in daily clinical practice among individuals with PA. Also, we point out on the value of replicating this study in a larger and fully matched sample size.

## Figures and Tables

**Table 1:** (**a**). General characteristics of the participants and the differences between the two groups. (**b**). Participants’ comorbidities. (**c**). Participants’s laboratory data.

	PA Group	EH Group	Statistical Test Results
(**a**)			
Systolic blood pressure (mmHg), mean (SD)	155.3 (17.9)	135.4 (12.2)	t(28) = 3.559, *p* = 0.001
Diastolic blood pressure (mmHg), mean (SD)	99.3 (13.1)	87.6 (4.9)	t(17,874) = 3.217, *p* = 0.005
Mean arterial pressure (mmHg), mean (SD)	117.9 (13.8)	103.5 (6.8)	t(20,342) = 3.629, *p* = 0.002
Duration of hypertension (years), median (Min–Max)	10 (1–22)	3 (1–17)	U = 164, *p* = 0.033
Number of medications, median (Min–Max)	3 (2–5)	4 (0–7)	U = 98, *p* = 0.551
Number of antihypertensive medications, median (Min–Max)	3 (2–5)	3 (0–6)	U = 126.5, *p* = 0.563
Number of participants with uncontrolled hypertension (N)	13	8	OR= 5.688, *p*= 0.108
BMI (kg/m^2^), mean (SD)	28.9 (4.6)	29.2 (6.1)	t(28) = −0.165, *p* = 0.870
Waist circumference (cm), mean (SD)	100.9 (17.3)	102.3 (13.4)	t(28) = −0.259, *p* = 0.797
Smokers (N)	2	2	OR = 1, *p* = 1
LVH (N)	4	4	OR = 1, *p* = 1
Education (yrs), median (Min–Max)	16 (4–16)	16 (9–17)	U = 76, *p* = 0.124
(**b**)			
Dyslipidaemia (N)	3	6	OR = 0.375, *p* = 0.427
DM2 (N)	0	1	OR = 0, *p* = 1
Hyperuricaemia (N)	0	1	OR = 0, *p* = 1
Urolithiasis (N)	3	1	OR = 3.5, *p* = 0.598
Controlled hypothyroidism (N)	1	2	OR = 0.464, *p* = 1
Controlled hyperthyroidism (N)	0	1	OR = 0, *p* = 1
Polycystic ovary syndrome (N)	1	0	OR = 0, *p* = 1
MGUS (N)	1	0	OR = 0, *p* = 1
Gastritis (N)	0	2	OR = 0, *p* = 0.483
Psoriasis (N)	0	1	OR = 0, *p* = 1
(**c**)			
Serum sodium (mmol/L), mean (SD)	142.7 (2.8)	140.8 (2.8)	t = 1.864, *p* = 0.073
Serum potassium (mmol/L), mean (SD)	3.6 (0.5)	4.3 (0.4)	t = −4.315, *p* < 0.001
Serum chloride (mmol/L), median (Min–Max)	102.5 (96–106)	104 (104–108)	U = 26, *p* = 0.073
Serum aldosterone (ng/dL), median (Min–Max)	28.6 (18.2–58)	11.6 (3.7–26.2)	U = 106, *p* = 0.002
Plasma renin activity (ng/mL/hr), median (Min–Max)	0.1 (0.1–0.5)	1.1 (0.5–1.6)	U = 1, *p* < 0.001
Aldosterone to renin ratio, median (Min–Max)	185.7 (43.8–580.4)	16.4 (3.1–25.8)	U = 120, *p* < 0.001
24 h urine sodium excretion (mmol/du), mean (SD)	206.8 (95.3)	222.1 (69.9)	t = −0.397, *p* = 0.696
24 h urine potassium excretion (mmol/dU), mean (SD)	88 (37.6)	74.1 (28.4)	t = 0.857, *p* = 0.402
24 h urine albumin excretion rate (mg/dU), median (Min–Max)	22 (5–330)	8 (5–39)	U = 122.5, *p* = 0.039

BMI—body mass index, DM2—type 2 diabetes, EH—essential hypertension, LVH—left ventricular hypertrophy, MGUS—monoclonal gammopathy of unknown significance, OR = odds ratio, PA—primary aldosteronism, SD—standard deviation.

**Table 2:** Cognitive testing results in the examined groups.

	PA Group	EH Group	Statistical Test Results
Adjusted MMSE (%), mean (SD)	98.3 (±8.2)	101.2 (±2.9)	t(17.42) = 1.304 *p* = 0.209
MoCA score (n), mean (SD)	25.1 (±2.2)	27.1 (±2.2)	t(28) = −2.544 *p* = 0.021

EH—essential hypertension, MMSE—Mini-Mental State Examination, MoCA—Montreal Cognitive Assessment, PA—primary aldosteronism, SD—standard deviation.

**Table 3:** Differences in cognitive function domains tested by the MoCA in the examined and control groups.

MoCA Test Categories	PA	EH	*p*-Value
Visuospatial/executive abilities—scores, median (Min–Max)	4 (2–5)	5 (3–5)	0.206
Naming—scores, median (Min–Max)	3 (3–3)	3 (3–3)	NaN
Attention and concentration—scores, median (Min–Max)	6 (3–6)	6 (5–6)	0.066
Language—scores, median (Min–Max)	2 (2–3)	3 (1–3)	0.085
Abstract thinking—scores, median (Min–Max)	2 (1–2)	2 (1–2)	0.479
Delayed recall—scores, median (Min–Max)	3 (2–5)	3 (0–5)	0.847
Orientation—scores, median (Min–Max)	6 (6–6)	6 (5–6)	NaN

EH—essential hypertension, MoCA—Montreal Cognitive Assessment, NaN—no variance between groups, PA—primary aldosteronism.

**Table 4:** Correlation analysis of primary aldosteronism participants’ characteristics and their cognitive function tests.

	Correlation Analysis Coefficient	Adjusted MMSE Score	MoCA Score
Serum aldosterone concentration (ng/dL)	Kendall’s Tau B	−0.128	−0.221
	*p*-value	0.517	0.268
ARR (ng/dL/ng/mL/h)	Kendall’s Tau B	−0.149	−0.176
	*p*-value	0.596	0.529
SBP (mmHg)	Pearson’s R	−0.108	−0.196
	*p*-value	0.701	0.484
DBP (mmHg)	Pearson’s R	−0.136	0.084
	*p*-value	0.629	0.766
MAP (mmHg)	Pearson’s R	−0.133	−0.031
	*p*-value	0.637	0.912
Serum potassium (mmol/L)	Pearson’s R	−0.224	0.134
	*p*-value	0.422	0.635
Serum sodium (mmol/L)	Pearson’s R	0.047	−0.219
	*p*-value	0.869	0.432
Fasting blood glucose (mmol/L)	Pearson’s R	−0.341	−0.621
	*p*-value	0.214	0.013
eGFR CKD EPI (mL/min/1.73 m^2^)	Pearson’s R	0.317	0.148
	*p*-value	0.250	0.598
24 h urine albumin excretion rate (mg/dU)	Kendall’s Tau B	−0.446	−0.254
	*p*-value	0.024	0.207
WC (cm)	Pearson’s R	0.016	−0.222
	*p*-value	0.955	0.427
BMI (kg/m^2^)	Pearson’s R	−0.004	−0.407
	*p*-value	0.988	0.132
Duration of hypertension (yrs)	Pearson’s R	−0.423	−0.495
	*p*-value	0.117	0.061
Antihypertensives (n)	Kendall’s Tau B	0.201	−0.036
	*p*-value	0.351	0.868
Total medications (n)	Kendall’s Tau B	0.201	−0.036
	*p*-value	0.351	0.868

ARR-aldosterone/renin ratio, BMI—body mass index, DBP—diastolic blood pressure, eGFR CKD EPI—estimated glomerular filtration rate calculated by CKD EPI equation, MAP—mean arterial pressure, MMSE—Mini-Mental State Examination, MoCA—Montreal Cognitive Assessment, SBP—systolic blood pressure, WC—waist circumference.

**Table 5:** Results of multiple linear regression analysis for MoCA test scores and predictor variables.

MODEL.	Variable	B	SE	β	t	*p*	95% CI
M_0_	Intercept	25.826	0.465		55.567	<0.001	[24.862, 26.790]
M_1_	Intercept	53.395	22.170		2.408	0.033	[5.090, 101.700]
	Aldosterone (ng/dL)	−0.158	0.066	−0.867	−2.409	0.033	[−0.302, −0.015]
	PRA (ng/mL/h)	4.276	2.059	0.956	2.077	0.060	[−0.210, 8.762]
	ARR (ng/dL/ng/mL/h)	0.009	0.005	0.622	1.921	0.079	[−0.001, 0.020]
	SBP (mmHg)	−0.007	0.029	−0.061	−0.257	0.802	[−0.070, 0.055]
	Duration of hypertension (years)	−0.005	0.074	−0.018	−0.072	0.944	[−0.167, 0.156]
	Education (years)	0.049	0.120	0.072	0.408	0.691	[−0.212, 0.310]
	Fasting blood glucose (mmol/L)	−1.016	0.644	−0.321	−1.577	0.141	[−2.420, 0.388]
	Serum sodium (mmol/L)	−0.165	0.146	−0.210	−1.132	0.280	[−0.484, 0.153]
	24 h urine albumin excretion rate (mg/dU)	−0.014	0.006	−0.442	−2.396	0.034	[−0.027, −0.001]
	Group (PA)	3.819	1.901		2.009	0.068	[−0.322, 7.960]

R2 = 0.721, F = 3.097, *p* = 0.034. ARR-aldosterone to renin ratio, PA—primary aldosteronism, PRA—plasma renin activity, SBP—systolic blood pressure, SE—standard error.

## Data Availability

The raw data supporting the conclusions of this article will be made available by the authors on request.

## Supplementary Materials

The following supporting information can be downloaded at: https://www.mdpi.com/article/10.3390/jcm14134618/s1, Table S1: Additional laboratory data of the participants.

## Abbreviations

The following abbreviations are used in this manuscript:

ARR	aldosterone/renine ratio
BMI	body mass index
CNS	central nervous system
DBP	diastolic blood pressure
eGFR CKD EPI	estimated glomerular filtration rate calculated by CKD EPI equation
EH	essential hypertension
HbA1c	haemoglobin A1c
Lp(a)	lipoprotein a
LVH	left ventricular hypertrophy
MAP	mean arterial pressure
MCI	mild cognitive impairment
MGUS	monoclonal gammopathy of unknown significance
MMSE	Mini-Mental State Examination
MoCA	Montreal Cognitive Assessment
MR	mineralocorticoid receptors
MRI	magnetic resonance imaging
OR	odds ratio
PA	primary aldosteronism
PRA	plasma renin activity
SBP	systolic blood pressure
SD	standard deviation
UHC	University Hospital Center
WC	waist circumference
WML	white matter lesion
