# Supplementary material for: Primary Aldosteronism and Cognitive Dysfunction: A Case-Control Study
Source: J Clin Med. 2025 Jun 30;14(13):4618. doi: 10.3390/jcm14134618 (PMC12251069; doi:10.3390/jcm14134618)
Supplement: Supplementary file 1 [file jcm-14-04618-s001.zip › jcm-3696018-supplementary.pdf]

Supplementary Table S1. Additional laboratory data of the participants

	**PA group**	**EH group**	**Statistical test results**
Haemoglobin (g/l), mean (SD)	148.5 (9.8)	144.8 (10.9)	t=0.875, p=0.389
Haematocrit (l/l), mean (SD)	0.427 (0.024)	0.426 (0.027)	t=0.014, p=0.989
Serum creatinine (umol/l), median (min-max)	77 (42-108)	69 (58-112)	U=135, p=0.360
eGFR CKD EPI (ml/min/1.73m^2), mean (SD)	89.3 (14.6)	92.7 (12.4)	t=-0.350, p=0.729
Serum urea (mmol/l), median (min-max)	5.5 (3.3-6.7)	5 (4-6.8)	U=140, p=0.262
Aspartate transaminase (U/l), median (min-max)	24 (14-33)	24 (18-25)	U=112.5, p=1
Alanine transaminase (U/l), mean (SD)	31.3 (11.8)	29.8 (5.7)	t=-0.243, p=0.810
Alkaline phosphatase (U/l), mean (SD)	71.9 (18.7)	69.9 (20.8)	t=-0.260, p=0.797
Gamma-glutamyltransferase (U/l), median (min-max)	27 (12-52)	24 (13-63)	U=78.5, p=0.856
Bilirubin (umol/l), mean (SD)	12.5 (5.6)	10.8 (2.6)	t=-0.869, p=0.394
Serum calcium (mmol/l), mean (SD)	2.3 (0.1)	2.3 (0.1)	t=0.649, p=0.524
Serum phosphorus (mmol/l), mean (SD)	0.9 (0.2)	1 (0.0)	t=1.196, p=0.248
Fasting blood glucose (mmol/l), median (min-max)	5.3 (4.2-6.7)	5.4 (4-9.3)	U=105, p=0.071
HbA1c (%), mean (SD)	5.5 (0.3)	5.5 (0.3)	t=0.171, p=0.867
Serum uric acid (mmol/l), mean (SD)	338.1 (90.3)	344 (110.2)	t=0.149, p=0.083
Total cholesterol (mmol/l), mean (SD)	4.8 (0.8)	4.9 (0.9)	t=0.253, p=0.803
LDL cholesterol (mmol/l), mean (SD)	2.8 (0.8)	2.9 (0.7)	t=0.436, p=0.997
Lp (a) nmol/l, median (min-max)	28 (7-69)	19.5 (6-134)	U=24, p=0.721

eGFR CKD EPI – estimated glomerular filtration rate calculated by CKD EPI equation, EH-essential hypertension, HbA1c-haemoglobin A1c, LDL – low density lipoprotein, Lp(a) – lipoprotein a, PA-primary aldosteronism
